# Genetic association between glucocorticoid receptor gene *Bcl1 rs41423247 and rs6198 polymorphisms* and risk of obesity in Egyptian children: By

**DOI:** 10.1038/s41598-025-94447-7

**Published:** 2025-04-08

**Authors:** Nanees A. Salem, Angi A. Alwakeel, Mayada Abdel-Latif, Shaimaa R. Hendawy, Mai S. Korkor

**Affiliations:** 1 Pediatric Department, Faculty of Medicine, Mansoura University, Mansoura, Egypt; 2 Clinical Pathology department, Faculty of Medicine, Mansoura University, Mansoura, Egypt; 3 Faculty of Medicine, Mansoura University, Mansoura, Egypt; 4 Resident of Pediatrics, Faculty of Medicine, Mansoura University Children’s Hospital, Mansoura University, Mansoura, Egypt; 5 Lecturer of Pediatrics, Faculty of Medicine, Mansoura University, Mansoura, Egypt

**Keywords:** Glucocorticoid, Bcl1 gene polymorphism, Obesity, Children, Genetics, Endocrinology

## Abstract

Obesity represents a major global public-health problem during childhood and adolescence. The genetic contribution to obesity and its consequences is well-established. Variation in glucocorticoid (GC)-sensitivity can be partly explained by polymorphisms in GC receptor (GR) gene where *NR3C1; Bcl1 rs41423247* and *NR3C1 rs6198* single nucleotide polymorphisms (SNPs) have been linked to higher and lower GC sensitivity, respectively. We aimed to explore the potential association between the GR gene SNPs and risk of obesity in a cohort of Egyptian children. We included 100 pre-pubertal children; 60 obese children and 40 age-and sex-matched normal-weight controls. *Bcl1 rs41423247* SNP was genotyped using PCR-restriction fragment length polymorphism technique and *NR3C1 rs6198* SNP was genotyped using Real-time-PCR.In *Bcl1 rs41423247*, obese children had more frequent CG, GG genotypes and G allele compared to healthy controls (*P* = 0.039, 0.019 and 0.007 respectively). Moreover, insulin resistance was significantly higher in combined CG + GG group compared to CC group. On the contrary, no significant differences were found in genotypes, alleles frequencies or insulin resistance between obese and non-obese children in *NR3C1 rs6198.* GR *Bcl1 rs41423247* gene polymorphism may play a role in genetic susceptibility to obesity that can be a future targeted therapy for obesity.

## Introduction

Obesity in children has become a global public-health problem that threatens their quality of life^1^. It increases the risk of type 2 diabetes, cardiovascular diseases, fatty liver, and certain types of cancer^2,3^. Hence, it is a leading cause of morbidity and mortality as 80% of the youth with obesity have obesity in their adulthood^4^.

Obesity prevalence is rising dramatically due to sedentary life and fast food consumption^5^. In addition, previous genetic studies have shown that the common polygenic obesity results from the interplay of genetic and environmental factors^6^. With the advent of genome-wide association studies (GWAS), hundreds of polymorphisms in genes and loci such as FTO, TMEM18, CADM1, CADM2, and NEGR1 have been associated with increased susceptibility to polygenic obesity^7^.

Moreover, polymorphisms in the nuclear receptor subfamily 3 group C member 1 (NR3C1) gene, encoding for the glucocorticoid receptor (GR), located on chromosome 5q31-32, encodes a nuclear transcription factor that mediates glucocorticoids (GCs) signaling are linked with obesity-related cardiovascular risk factors^8^.

The *NR3C1 (BclI*,* rs41423247)* polymorphism, is a C > G substitution, located in intron 2, on chromosome 5q31.3. This C > G single nucleotide polymorphism (SNP) has been associated with increased sensitivity to GCs and with increased body mass index, body fat centralization ratio, dyslipidemia, insulin resistance, endothelial dysfunction and inflammation^8–12^. Thus, it contributes to obesity phenotype throughout all age categories from prepubertal children to adolescents, adults and in the elderly^19,20^. However, the underlying molecular mechanism is still not completely understood. It could be attributed to increased sensitivity of negative feedback mechanism of GCs or possible linkage disequilibrium that can alter the transcriptional activity of target genes involved in glucose and insulin homeostasis^19^.

Additionally, another *NR3C1* polymorphism (*rs6198*), is an A > G substitution at nucleotide position *3669* base pair (bp) (*A3669G*), located in the 3′ untranslated region of the alternative spliced exon 9β, on chromosome 5q31.3, has been linked with relative GCs resistance. Recent studies have suggested that *A3669G* carriers have an increased pro-inflammatory state and an increased risk for cardiovascular disease^8,13^. Moreover, this A > G SNP was associated with binge eating disorder characterized by uncontrolled hyperphagia till the sense of discomfort and excess fullness that can contribute to even morbid obesity^21^.

Few studies have investigated the relation between polymorphisms in the GCR gene and body mass index, blood pressure and cholesterol in obese elderly patients^8^, but no such studies involving pediatric age group. *The aim of the current pilot study* was to explore for the first time the potential genetic association between GR gene polymorphisms, *NR3C1; Bcl1 rs41423247* and *NR3C1 rs6198*, and risk and severity of obesity in children.

## Subjects and methods

**Study designs: **This is a cross-sectional, case control study.

**Participants: **100 participants: 60 obese children and 40 healthy controls. Obese children were recruited during their follow up at outpatient endocrine clinic, Mansoura University Hospital, Egypt during the year 2020–2022. Obese children were subclassified into three classes: moderate (*n* = 20), severe (*n* = 20), and morbid (*n* = 20) obesity. Normal weight-healthy controls were recruited from a primary school in the same locality. Overall, participants belong to middle-class families, and none reported participation in organized physical activity.

**Inclusion criteria: **(a) prepubertal children aged from 4 to 11 years (b) obesity defined as BMI more than 95th percentile for age and gender (c) patients on regular follow up. **Exclusion criteria**: Patients who had syndromic obesity, dysmorphic features, drug induced obesity as prolonged steroid therapy, current infections, chronic comorbidities, irregular follow up visits, or refused to be enrolled in the study were excluded from the study. Obese children were subclassified into three classes: moderate (*n* = 20), severe (*n* = 20), and morbid (*n* = 20) obesity^14^.

**Data collection: **Socio-demographic as age, gender, parents’ education and occupation, residence and socio-economic standard, activity out, sleep hours, TV watching hours and appetite were collected.

### Clinical evaluation

Examination was done by a single well-trained nurse to avoid interindividual variations and risk of bias. The body weight and height of participants were measured according to standardized techniques. The body mass index (BMI) was calculated by dividing weight (kg) by height squared (m2). Waist circumference (WC) was measured at the midpoint between the lowest ribs and the iliac crests at the end of normal expiration. Mid-thigh circumference (MTC) was measured in centimeters (cm) on the right thigh at mid-point between the top of the femur and the knee while the child was standing, while neck circumference (NC) was measured in cm while the subjects standing upright at mid-point between mid-cervical spine and mid anterior neck, using non-stretchable plastic tape. For all participants, weight, height, and BMI Z-scores were calculated using reference data for Egyptian children^15^.

## Definition of obesity & obesity classes

Children are considered obese when their BMI ≥ 95th percentile for their age and gender. Obese Children were further classified into three obesity classes based on the percentage of BMI above the 95th BMI percentile; moderate obesity (%BMI ≥ 100%); severe obesity (%BMI ≥ 120%), and morbid obesity (%BMI ≥ 140%)^16,17^.

Systolic and diastolic blood pressure (SBP/DBP) was obtained using conventional mercury sphygmomanometer following standard technique^18^. Pubertal development was determined in all participants using Tanner classifications^19^. We included prepubertal children only to investigate the study variables independent of the impact of sex steroids.

### Body composition measurements

Body composition measurements were obtained by bioimpedance technique using Tanita BC-418MA body composition analyzer (Tanita Corp., Tokyo, Japan). We strictly followed the instructions provided by the manufacturer. Body adiposity indicators include percentage of total body fat (TB-F%), total body-fat mass (TB-FM; kg), trunk-FM (kg), a marker of central adiposity, and appendicular-FM (kg) as a sum of the FM in the four limbs that reflect peripheral fat distribution. Trunk-FM to appendicular-FM ratio was calculated^20^.

### Biochemical evaluation

Blood samples were aseptically collected in early morning from all participants, after a 12-hour overnight fast. Each blood sample divided into 1 ml of whole blood was collected on K2 EDTA tube (Greiner Bio One) for DNA extraction, and another sample for immediate serum separation and stored at − 20 °C until further use for metabolic panel analysis.

Fasting lipid profile including serum total cholesterol (TC) and triglycerides (TGs) were measured by colorimetric kit (Spinreact, Girona, Spain). High-density lipoprotein cholesterol (HDL-C) was measured by colorimetric kit (Human Diagnostics, Wiesbaden, Germany) and Low-density lipoprotein cholesterol (LDL-C) was estimated using the modified Friedewald’s formula. Serum fasting blood glucose (FBG) was measured by end point colorimetric reagents supplied by SPINREACT (S.A./S.A. U Ctra. Santa Coloma, 7 E-17176 SANT ESTEVE DE BAS (GI) SPAIN). Serum insulin was measured by quantitative sandwich ELISA (Human Insulin Kit (ab200011), abcam, USA). Serum adrenocorticotropic hormone (ACTH) was measured by electrochemiluminescence immunoassay.

### Definition of insulin resistance

**Insulin resistance** was assessed by Homeostasis model assessment of insulin resistance (HOMA-IR) as proposed by Matthews et al.^21^ using this formula: HOMA-IR = FBG (mmol/L) x Fasting Insulin (µU/mL) / 22.5. A HOMA-IR value above 2.5 was the cut-off point to determine insulin resistance in prepubertal children that corresponds to the 90th percentile of healthy children as mentioned in previous studies^22^.

### Definition of metabolic syndrome (MetS)

Children with obesity are considered to have MetS according to Adult Treatment Panel III (ATP III) criteria modified for the pediatric age group^23^ when they have at least three of the following five components;^1^ central adiposity (WC ≥ 90th percentile for age and gender);^2^ FBG ≥ 5.6 mmol/L (100 mg/dL);^3^ Triglycerides ≥ 1.7 mmol/L (150 mg/dL);^4^ HDL-C ≤ 1.03 mmol/L (40 mg/dL); and^5^ SBP and/or DBP ≥ 90th percentile for age, gender and height percentile.

### Single nucleotide polymorphism (SNP) genotyping

Genomic DNA was extracted from whole blood according to the manufacturer instructions (Gene JET Genomic DNA) Purification Kit; Thermo Scientific, EU Lithuania) and then was stored at − 20 °C until use.

*For NR3C1*,* Bcl1 rs41423247* (GR sensitivity), SNP was detected using the polymerase chain reaction -restriction fragment length polymorphism method (PCR-RFLP) using *Bcl1* restriction endonuclease (Roche Applied Science, Mannheim, Germany). We used F–5_TGCTGCCTTATTTGTAAATTCGT − 3 as a forward primer and 5 R–5_AAGCTTAACAATTTTGGCCATC − 3 as a reverse primer. The PCR protocol included 5 min of denaturation at 95 °C (1 cycle), followed by 1 min of denaturation at 94 °C (30 cycles). Then, annealing at 55 °C for 1.5 min (except for rs6195 polymorphism [exon 2–5] at 51 °C), extension at 72 °C for 1.5 min, and a final cycle for 5 min at 72 °C. RFLP analysis was performed to determine genotype frequencies, 5mL of relevant PCR product (335 bp) was digested by 10 units of rs41423247 restriction endonuclease (Roche Applied Science, Mannheim, Germany) for 3–16 h at 37 °C. Bands of 117, 222 bp correspond to *CC* genotype, three fragments (117, 222, and 335 bp) represent the *CG* genotype and single band of 335 bp represent the *GG* genotype^24^.

*For NR3C1*,* rs6198* (GR resistance), SNP was detected by Real-time PCR using TaqMan SNP Genotyping Assays (Applied Biosystems; Thermo Fisher Scientific). We utilized TaqMan Universal PCR Master Mix at *NR3C1 rs6198* loci using Step One Real-Time. The protocol mixed DNA template (20 ng) with a TaqMan SNP Genotyping Assay Mix (1 µL) with TaqMan Universal PCR Master Mix (10 µL) resulting in a 20 µL final volume. We used the proper negative controls in each run^25^.

Analysis of studied SNPs genetic features was performed according to National Center for Biotechnology Information (NCBIs).

### Statistical analysis

The SPSS software (Statistical Package for the Social Sciences, version 25, SPSS Inc, Chicago, Ill, USA) was used for data analysis. Mean and Standard Deviation (SD) or median and interquartile range (IQR) represent quantitative data whereas numbers (N) and percentages (%) represent qualitative data. The Student T test and Mann Whitney Test (U test) were used for 2 groups comparison for parametric quantitative and non-parametric variables, respectively. The Kruskal Wallis test was used to compare nonparametric variables between multiple groups analysis. For qualitative variables, the Chi-Square test was used to compare two groups and Fisher’s exact test when the expected count is less than 5 in more than 20% of cells. The strength of the associations was estimated by Odds ratio (OR) and 95% confidence interval (CI). Deviation from Hardy– Weinberg equilibrium (HWE) for the studied genes were evaluated by comparing the observed and expected genotype frequencies in the control group. There was no deviation from HWE, with p values 0.763 and 0.944 for rs41423247 and rs6198, respectively. Statistically significant difference was considered when the P value < 0.05.

## Results

### Sample size calculation

The calculated sample size of the study was sixty participants at 5% level of significance and 80% power of the study, using G*Power 3 sample size calculator. Genotypes frequencies in the group with obesity = 26%, Genotypes frequencies in control group = 9%^26^. Using exact test, inequality, two independent groups, with a power of 80% and alpha error of 5%, the required minimal sample size was at least 32 per group. The sample size was increased to increase the power of the study, cases group size was increased to 60, while control group was increased to 40 subjects.

Matching of controls to cases was done using “frequency matching”. The controls were not individually matched to specific cases, but the overall distribution of controls was chosen to match the distribution of cases based on age and sex.

**Fig. 1:**
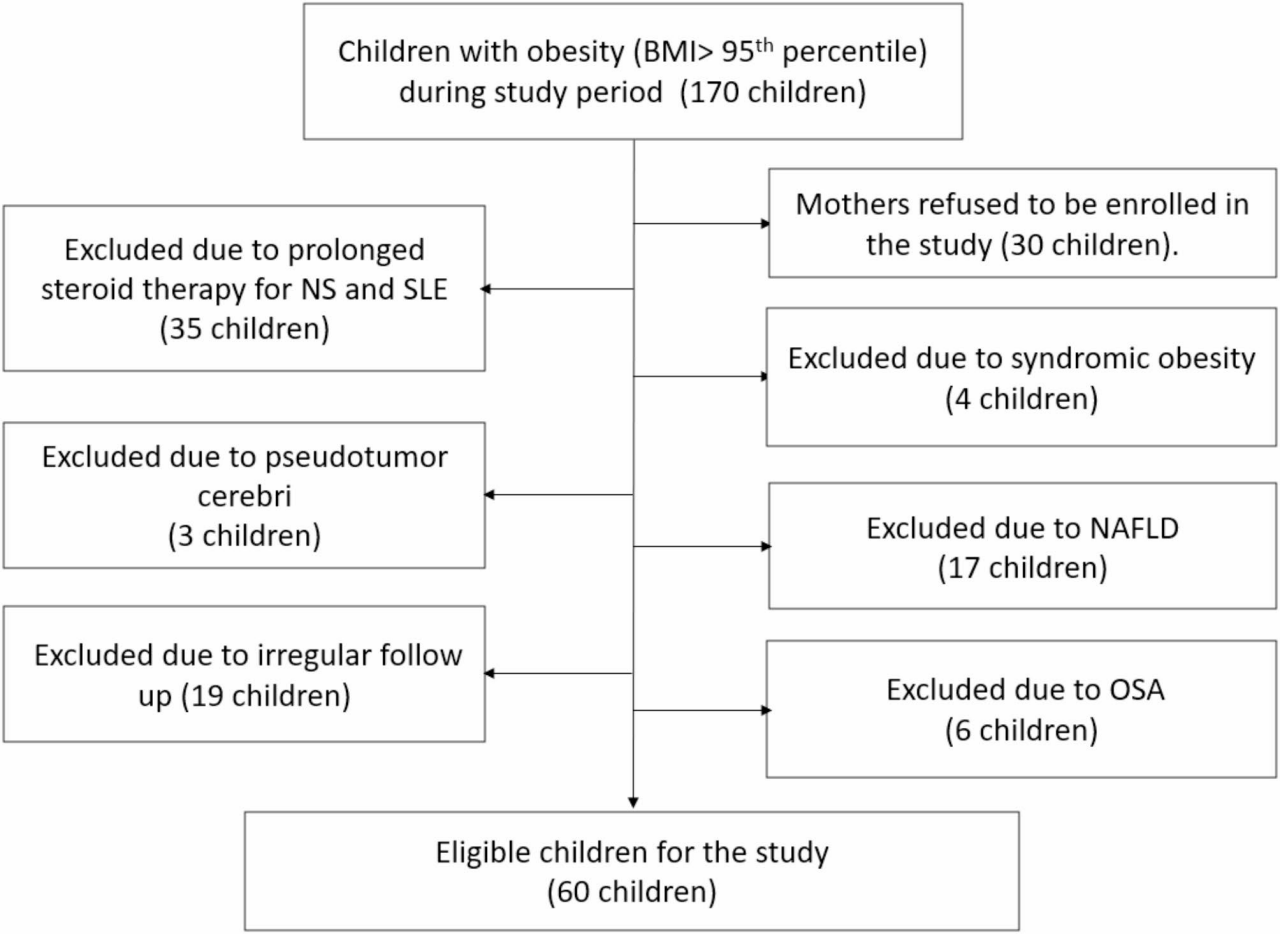
Flow chart of study participants in the present study. BMI: body mass index, NS: nephrotic syndrome, SLE: systemic lupus erythematosus, NAFLD: non-alcoholic fatty liver disease, OSA: obstructive sleep apnea.

Thus, the study included 100 prepubertal children (4–11 years); 60 obese children who fulfilled selection criteria as illustrated in the flow chart (Fig. 1) and 40 normal-weight healthy controls. The two groups were age-and sex-matched (*p* = 0.705 and *p* = 1.00 receptively). The family history of obesity was detected in 95.0% of obese children and 61.7% of parents were educated. They have a median of one-hour outdoor activity per day and sleeping around 9 h per day and TV watching around 3 h per day.

Demographics, clinical data, anthropometric measurements, and biochemical characteristics of studied groups are summarized in Table 1. All participants were normotensive. Obese children had higher blood pressure, anthropometric measurements and dyslipidemia compared to healthy controls. Moreover, obese children had higher FBG, fasting insulin, and HOMA-IR values and higher incidence of IR and MetS compared to control.

Allelic frequencies of *BclI rs41423247* and *rs6198* polymorphisms were in Hardy-Weinberg equilibrium (P-values > 0.05).

In *Bcl*1 *rs41423247* SNP, obese children had significantly higher GG, CG genotypes and *G* allele compared to healthy controls. However, no significant differences were found between obese children and healthy controls in both genotypes and allelic frequencies in *rs6198* SNP, as illustrated in Table 2.

Anthropometric and body composition parameters of three obesity class subgroups are illustrated in Table 3. Children with morbid obesity had higher weight Z, BMI, BMI-Z, Fat %, FM, trunk FM, and appendicular FM compared to those with moderate and severe obesity.

Analysis of the genotypes and alleles frequencies in the obesity subgroups revealed that no significant differences were found in both studied SNPs as in Table 4.

There were significantly higher BMI, fat mass, insulin levels, HOMA-IR, and insulin resistance in combined CG + GG group compared to *CC* group in *NR3C1*,* Bcl1 rs41423247*. However, no significant difference was found between different genotypes of *NR3C1 rs6198*, as illustrated in Table 5.

## Discussion

Genetics play a vital role in the development of childhood obesity together with environmental and behavioural factors^27^. In this study, we investigated the association between Glucocorticoid receptor; *NR3C1; Bcl1 rs41423247* and *rs6198* Gene Polymorphism and childhood obesity. To the best of our knowledge, it is the first Egyptian study done in obese children and adolescents on these two genes polymorphism. The study involved sixty children and adolescents with obesity and forty age and sex matched healthy controls.

In the present study, obese children had significantly higher anthropometric measurements, blood pressure and laboratory markers of dyslipidaemia. Out of the sixty obese children, twenty-nine children fulfilled the criteria for metabolic syndrome diagnosis based on Adult Treatment Panel III (ATP III) criteria modified for the pediatric age groups. Moreover, children with morbid obesity had higher insulin levels and HOMA-IR than those with moderate and severe obesity.

Regarding ***Bcl1 rs41423247***; obese children had significantly higher *GG*,* CG* genotypes and *G* allele than the healthy controls. This may indicate that having at least one *G* allele can be considered a risk factor to develop obesity. Additionally, there was significant elevation of insulin levels and HOMA-IR in combined *CG + GG* groups compared to *CC* group. However, no significant difference was found in genotypes and alleles frequency between obesity subgroups (moderate, severe, and morbid ones). This can be explained by small number of cases in each group.

In line with our results, another study by Castellini et al., found that ***Bcl1 rs41423247*** GG and GC genotypes were associated with Emotional disorders and hyperphagia evaluated by Emotional Eating Scale " EES”^28^. This can be attributed to GR hyper-function that can lead to emotional dysregulation and HPA-axis dysregulation and inhibition of the regulatory feedback.

Moreover, ***Bcl1 rs41423247*** GR has been associated with enhanced glucocorticoids sensitivity and consequently higher BMI, waist to hip ratio, higher blood pressure, worse metabolic profile hyperinsulinemia, impaired glucose tolerance and dyslipidaemia^29^. In another large study, performed by Geelen et al., on 1228 adults with obesity, they found that *GG* genotype had higher BMI, WC and HMOA IR than *CG* and *CC* genotypes^30^. This was explained by increased receptors sensitivity and consequently adverse effects of endogenous GCs. Another study in children revealed that the *GG* genotype was associated with increased whole-body fat mass and fat mass distribution in prepubertal obese children using dual-energy x-ray absorptiometry^31^.

It was also reported in another study, performed on 742 individuals, that *BclI* gene SNP is associated with higher amount of abdominal visceral fat deposition (assessed by computed tomography) independent of the level of total body fat (assessed by hydro densitometry). Thus, it can contribute to abdominal obesity and development of metabolic syndrome^32^.

Moreover, Di Blasio and colleagues investigated the role of *N363S* and *BclI* polymorphisms in 279 elderly Italian patients with severe obesity (mean BMI 45·9 ± 0·9 kg/m2). They identified thirteen heterozygotes for *N363S* allele who had significantly higher BMI, resting energy expenditure and food intake when compared to wild-type homozygotes. In addition, the allele frequency of *BclI* variant was 27% with no detected significant differences in anthropometric and metabolic parameters between heterozygous or homozygous subjects for this variant in this obese population. Furthermore, they identified that subjects who carried both polymorphisms had a tendency to have a slightly unfavourable cardiovascular profile with higher systolic and diastolic blood pressure and significantly higher total and LDL cholesterol levels^29^.

In addition, it was observed that GG genotype of *BclI gene* SNP was associated with higher BMI and higher ratio of fat centralization in patients with bronchial asthma than healthy controls^33^. In another study, *CG* genotype had increased tendency to accumulate adipose tissue in comparison to both *GG* and *CC* genotypes^34^. This discrepancy may be related to ethnic variation.

The association between Bcl-1 polymorphism and increased susceptibility to obesity can be explained through the alteration of GR sensitivity to endogenous steroids. This can be mediated through direct effect of Bcl-1 polymorphism on GR gene expression or through its influence on the transcriptional or trans-repression activity of the targeted genes involved in glucose and insulin homeostasis^35,36^. Transrepression means interaction with transcriptional factors that prevent their binding to the target genes representing an indirect way of regulation of the GR gene expression in the absence of direct DNA binding^37,38^. Thus, GR polymorphisms have a crucial role in the development of obesity and metabolic syndrome^39^.

In the contrary to our results, Buemann et al., concluded that *BclI* gene SNP has no association with BMI, fat percentage or abdominal obesity. However, it had significant association with abdominal visceral fat adjusted for total body fat mass. The impact on visceral adiposity was uncovered only after statistical adjustment for the effect of total body fat. Thus, GRL gene SNP may influence the body fat distribution^10^.

Regarding *NR3C1 rs6198* SNP, there was no significant difference between different genotypes and allele frequency between obese children and healthy children or between different subgroups with obesity. In contrary to our result, another study by Cellini et al., revealed that *NR3C1 rs6198* SNP was associated to binge eating symptoms, whereas no significant association was found between *Bcl1 rs41423247* and the other eating disorders^40^. The points of strength of the current study include being in a peculiar age group of Egyptian population. However, it is limited by relatively small sample size and being a single center study.

## Conclusion

Obese children had higher CG, GG genotypes and *G* allele of *Bcl1 rs41423247* gene SNP and this can be attributed to its effect on the GR. This can be a future target therapy for obesity in children to avoid its deleterious complications.

**Table 1:** Demographics, clinical data, anthropometric measurements, and biochemical characteristics of studied groups.

Variables			Control group (*n* = 40)	Group with obesity (*n* = 60)	*P* value
Age		median (IQR)	9.7 (4–13)	9.7 (4–12)	0.705
Gender	Male	N (%)	16 (40.0%)	24 (40.0%)	1.00
	Female	N (%)	24 (60.0%)	36 (60.0%)	
Residence	Urban	N (%)	14 (35.0%)	13 (21.7%)	0.141
	Rural	N (%)	26 (65.0%)	47 (78.3%)	
Obesity grades	Moderate	N (%)		20 (33.3%)	
	Severe	N (%)		20 (33.3%)	
	Morbid	N (%)		20 (33.3%)	
SBP (mmHg)		median (IQR)	100.0 (90.0-140.0)	110.0 (90.0-140.0)	< 0.001*
DBP (mmHg)		median (IQR)	65.0 (60.0–70.0)	70.0 (60.0-100.0)	< 0.001*
Anthropometric measurements					
Weight (Kg)		median (IQR)	28.1 (15.8–50.0)	63.6 (22.4–97.0)	< 0.001*
Weight Z		median (IQR)	-0.700 (-1.5-1.7)	3.7 (1.9-9.0)	< 0.001*
Height (cm)		median (IQR)	131.0 (108–150)	139.0 (94–155)	0.015*
Height Z		median (IQR)	-0.175 (-1.9-1.8)	0.9 (-1.7-3.5)	< 0.001*
BMI (kg/m2)		median (IQR)	15.8 (12.4–22.2)	32.2 (18.8–43.3)	< 0.001*
BMI Z		median (IQR)	-0.6 (-1.6-1.14)	3.7 (2.2–8.5)	< 0.001*
BMI %		median (IQR)	63.8 (43.6–92.5)	126.7 (103.0-167.0)	< 0.001*
WC (cm)		median (IQR)	69.0 (44.0–84.0)	95.0 (50.0-140.0)	< 0.001*
MTC (cm)		median (IQR)	37.5 (27.0–65.0)	53.0 (39.0-100.0)	< 0.001*
NC (cm)		Mean ± SD	25.5 ± 3.27	34.75 ± 4.27	< 0.001*
Body Composition parameters					
BMR (Kcal)		median (IQR)	1113.0 (822–1336)	1532.0 (949–2284)	< 0.001*
Fat %		median (IQR)	20.0 (13.9–28.5)	40.5 (20.1–54.5)	< 0.001*
FM (Kg)		median (IQR)	4.90 (2.6–11.3)	26.9 (5.8–57.6)	< 0.001*
TB-MM (Kg)		median (IQR)	22.5 (13.2–31.6)	36.05 (14.7–55.0)	< 0.001*
Trunk FM (Kg)		median (IQR)	1.95 (1.1–5.5)	11.15 (2.5–23.1)	< 0.001*
Appendicular FM (Kg)		median (IQR)	3.0 (1.4-8.0)	15.2 (3.0-37.6)	< 0.001*
Trunk FM/ appendicular FM		Mean ± SD	0.684 ± 0.106	0.754 ± 0.149	0.012*
FM/TB-MM		median (IQR)	0.228 (0.16–0.4)	0.668 (0.25–1.19)	< 0.001*
Biochemical characteristics					
Serum cholesterol (mg/dl)		Mean ± SD	151.48 ± 17.26	172.94 ± 33.01	< 0.001*
Serum TG (mg/dl)		median (IQR)	97.0 (47.0-130.0)	116.0 (54.0-356.0)	0.003*
Serum HDL (mg/dl)		median (IQR)	50.0 (41.0–71.0)	47.0 (29.0–71.0)	0.003*
Serum LDL (mg/dl)		Mean ± SD	81.8 ± 17.1	102.4 ± 34.0	0.001*
FBG (mg/dl)		median (IQR)	90.0 (72–112)	100.0 (72–120)	0.007*
Fasting insulin (uIU/ml)		median (IQR)	8.95 (2.1–12.0)	14.4 (2.1–42.5)	< 0.001*
HOMA IR		median (IQR)	1.97(0.54–2.43)	3.50 (0.62–9.86)	< 0.001*
Insulin resistance		N (%)	0 (0.0%)	41 (68.3%)	< 0.001*
Metabolic syndrome		N (%)	0 (0.0%)	29 (48.3%)	< 0.001*

Mann-Whitney, independent T test and Chi-square tests were used, BMR: basal metabolic rate, BMI: Body mass index, DBP: Diastolic blood pressure, FBG: fasting blood glucose, FM: fat mass, HDL: high density lipoproteins, Homeostatic Model Assessment for Insulin Resistance, IQR: interquartile range, LDL: low density lipoproteins, MTC: Mid-thigh circumference NC: Neck circumference, SBP: Systolic blood pressure, TG: triglycerides, WC: Waist circumference, *Significant P value < 0.05.

**Table 2:** Distribution of *NR3C1; Bcl1 rs41423247* and *rs6198* genotypes and alleles in obese children versus controls.

			Group with obesity (*n* = 60) N (%)	Control group (*n* = 40) N (%)	Relative risk of obesity	
					OR (95%CI)	P value
*NR3C1; Bcl1 rs41423247*	Genotypes	CC	15 (25.0%)	20 (50.0%)	Reference	-
		CG	31 (51.7%)	16 (40.0%)	2.583 (1.049–6.361)	0.039*
		GG	14 (23.3%)	4 (10.0%)	4.666 (1.275–17.077)	0.019*
		CG + GG	45 (75.0%)	20 (50.0%)	3.00 (1.279–7.031)	0.011*
	Alleles	C	61 (50.8%)	56 (70.0%)	2.256 (1.241–4.101)	0.007*
		G	59 (49.2%)	24 (30.0%)		
*NR3C1 rs6198*	Genotypes	AA	27 (45.0%)	19 (47.5%)	Reference	-
		AG	24(40.0%)	17(42.5%)	0.993 (0.422–2.335)	0.988
		GG	9(15.0%)	4(10.0%)	1.583 (0.424–5.903)	0.493
		AG + GG	33(55.0%)	21(52.5%)	1.105 (0.495–2.466)	0.805
	Alleles	A	78(65.0%)	55(68.8%)	1.184 (0.647–2.166)	0.582
		**G**	42(35.0%)	25(31.2%)		

A: adenine, C: cytosine, G: Guanine, Logistic regression analysis test was used, Reference genotype and allele according to NCBI, CI: confidence interval, OR: odds ratio, OR < 1 is considered protective and OR > 1 is considered risky, *significant: P value < 0.05.

**Table 3:** Comparison of anthropometric and body composition parameters of subgroups with obesity.

Variables		Moderate obesity (*n* = 20)	Severe obesity (*n* = 20)	Morbid obesity (*n* = 20)	*P* value
Anthropometric measurements					
Weight (Kg)	median (IQR)	56.7 (25.2–80.0)	65.6 (22.4–88.1)	71.0 (35.7–97.0)	0.040*
Weight Z	median (IQR)	2.8 (1.9–4.2)	3.55 (2.0-5.4)	5.22 (3.2-9.0)	≤ 0.001*
Height (cm)	median (IQR)	141.0 (109–155)	136.0 (94–155)	138.0 (105–150)	0.841
Height Z	median (IQR)	1.01 (-1.2-3.5)	0.88 (-1.7-1.9)	0.86 (-1.2-3.37)	0.443
BMI	median (IQR)	28.2 (18.8-34.89)	33.45 (24.0-39.2)	35.8 (29.9–43.3)	≤ 0.001*
BMI Z	median (IQR)	2.65 (2.2–3.90)	3.90 (2.0-5.50)	5.4 (2.80–8.50)	≤ 0.001*
BMI %	median (IQR)	111.0 (103–124)	126.7 (120-138.5)	143.0 (134–167)	≤ 0.001*
WC (cm)	median (IQR)	91.5 (70–116)	93.0 (50–114)	100.5 (62–140)	0.080
MTC (cm)	median (IQR)	52.5 (41–87)	52.5 (39–100)	58.0 (49–91)	0.175
NC (cm)	Mean ± SD	33.4 ± 4.17	34.45 ± 4.13	36.4 ± 4.17	0.077
Body composition parameters					
BMR (Kcal)	median (IQR)	1447.5 (949–1938)	1465 (1017–2000)	1637 (1041–2284)	0.209
Fat %	median (IQR)	37.0 (20.1–50.8)	38.7 (30.4–54.3)	45.9 (31.3–54.5)	≤ 0.001*
FM (Kg)	median (IQR)	20.8 (5.8–36.2)	27.0 (7.7–57.6)	30.7 (14.3–52.4)	0.006*
TB-MM (Kg)	median (IQR)	34.9 (18.5–50.0)	33.8 (14.7–50.0)	38.8 (21.4–55.0)	0.560
Trunk FM (Kg)	median (IQR)	9.1 (2.5–15.1)	11.7 (4.1–19.5)	12.5 (6.0 -23.1)	0.035*
Appendicular FM (Kg)	median (IQR)	12.0 (3.4–20.6)	15.1 (3.0-37.6)	20.4 (8.0–34.0)	0.004*
Trunk/appendicular FM	Mean ± SD	0.784 ± 0.080	0.785 ± 0.193	0.693 ± 0.140	0.082
TFM/TB-MM	median (IQR)	0.585 (0.25–1.03)	0.632 (0.44–1.19)	0.849 (0.45–1.19)	0.001*

Kruskal Wallis test was used, BMI: Body mass index, FM: fat mass, IQR: interquartile range, MTC: Mid-thigh circumference NC: Neck circumference, WC: Waist circumference, *Significant P value < 0.05, P, between 3 groups.

**Table 4:** Distribution of *Bcl1 rs41423247* and *NR3C1 rs6198* genotypes and alleles frequencies in subgroups with obesity.

		Moderate obesity (*n* = 20) N (%)	Sever obesity (*n* = 20) N (%)	Morbid obesity (*n* = 20) N (%)	RR of sever obesity		RR of morbid obesity	
					OR (95%CI)	P value	OR (95%CI)	P Value
*NR3C1; Bcl1 rs41423247*	CC	7 (35.0%)	4 (20.0%)	4 (20.0%)	Reference	-	Reference	-
	CG	10 (50.0%)	10 (50.0%)	11 (55.0%)	1.75 (0.386–7.91)	0.46	1.925 (0.430–8.860)	0.39
	GG	3 (15.0%)	6 (30.0%)	5 (25.0%)	3.50 (0.549–22.30)	0.18	2.916 (0.442–19.23)	0.26
	CG + GG	13 (65.0%)	16 (80.0%)	16 (80.0%)	2.153 (0.515- 9.00)	0.29	2.153 (0.515- 9.00)	0.29
	C	24 (60.0%)	18 (45.0%)	19 (47.5%)	1.83 (0.754–4.45)	0.18	1.657 (0.684–4.02)	0.26
	G	16 (40.0%)	22 (55.0%)	21 (52.5%)				
*NR3C1 rs6198*	AA	11 (55.0%)	8 (40.0%)	8 (40.0%)	Reference	-	Reference	-
	AG	4 (20.0%)	10 (50.0%)	10 (50.0%)	3.437 (0.786–15.01)	0.10	3.437 (0.78–15.01)	0.10
	GG	5 (25.0%)	2 (10.0%)	2 (10.0%)	0.550 (0.084–3.589)	0.53	0.550 (0.084–3.589)	0.53
	AG + GG	9 (45.0%)	12 (60.0%)	12 (60.0%)	0.550 (0.084–3.589)	0.53	0.55 (0.084–3.589)	0.53
	A	26 (65.0%)	26 (65.0%)	26 (65.0%)	1.00 (0.399–2.506)	1.00	1.00 (0.399–2.506)	1.00
	G	14 (35.0%)	14 (35.0%)	14 (35.0%)				

RR: relative risk, A: adenine, C: cytosine, G: Guanine, Logistic regression analysis test was used, Reference genotype and allele according to NCBI, CI: confidence interval, OR: odds ratio, OR < 1 is considered protective and OR > 1 is considered risky, *significant: P value < 0.05.

**Table 5:** Association between *Bcl1 rs41423247* and *NR3C1 rs6198* genotypes and alleles frequencies with the studied clinical and biochemical variables in the group with obesity.

Variables			CC group (*n* = 15)	CG + GG group (*n* = 45)	*P* value
*NR3C1; Bcl1 rs41423247*	BMI	Median (IQR)	29.0 (18.8–39.2)	33.3 (21.2–43.3)	0.038*
	Fat (kg)	Median (IQR)	20.2 (5.8–39.0)	28.3 (6.7–57.6)	0.014*
	Insulin (uIU/ml)	Median (IQR)	9.5 (2.1–23.0)	14.8 (3.4–42.5)	0.021*
	HOMA IR	Median (IQR)	1.88 (0.62–5.17)	3.65 (1.0-9.86)	0.017*
	IR	Median (IQR)	7 (46.7%)	34 (75.6%)	0.037*
	Metabolic syndrome	N (%)	6 (40.0%)	23 (51.1%)	0.456
Variables			AA group (*n* = 27)	AG + GG group (*n* = 33)	P value
*NR3C1* rs6198	BMI	Median (IQR)	30.7 (18.8–43.3)	33.9 (21.2–43.1)	0.345
	Fat (kg)	Median (IQR)	26.4 (5.8–46.5)	26.9 (6.7–57.6)	0.582
	Insulin (uIU/ml)	Median (IQR)	14.2 (3.4–42.5)	14.7 (2.1–35.2)	0.652
	HOMA IR	Median (IQR)	3.51 (1.0-9.86)	3.50 (0.62–7.01)	0.818
	IR	Median (IQR)	16 (59.3%)	25 (75.8%)	0.172
	Metabolic syndrome	N (%)	15 (55.6%)	14 (42.4%)	0.311

A: adenine, C: cytosine, G: Guanine, Mann-Whitney, independent T test and Chi-square tests were used, BMI: Body mass index, FM: fat mass, HOMA-IR: Homeostatic Model Assessment for Insulin Resistance, IQR: interquartile range, *Significant P value < 0.05.

## Electronic supplementary material

Below is the link to the electronic supplementary material.

Supplementary Material 1

## Data Availability

The datasets generated during and/or analysed during the current study are available from the corresponding author on reasonable request.
