# Supplementary material for: Genetic association between glucocorticoid receptor gene Bcl1 rs41423247 and rs6198 polymorphisms and risk of obesity in Egyptian children: By
Source: Sci Rep. 2025 Apr 8;15:12012. doi: 10.1038/s41598-025-94447-7 (PMC11978808; doi:10.1038/s41598-025-94447-7)
Supplement: Supplementary file 1 — Supplementary Material 1 [file 41598_2025_94447_MOESM1_ESM.pdf]

Genetic Association between Glucocorticoid Receptor Gene Bcl1 rs41423247 and rs6198 Polymorphisms and Risk of Obesity in Egyptian Children

By

Nanees A. Salem[1], Angi A. Alwakeel [1], Mayada Abdel-Latif [1], Shaimaa R. Hendawy [2], Mai S Korkor [1*].

[1]Pediatric Department, Faculty of Medicine, Mansoura University, Mansoura, Egypt.

[2]Clinical Pathology department, Faculty of Medicine, Mansoura University, Mansoura, Egypt.

Type of article: Original Article**.**

Title: Genetic Association between Glucocorticoid Receptor Gene Bcl1 rs41423247 and rs6198 Polymorphisms and Risk of Obesity in Egyptian Children

Running title: Glucocorticoid Receptor Gene Polymorphisms and Obesity in Children.

Author list:

Nanees A. Salem; Professor of Pediatrics, Faculty of Medicine, Mansoura University, Mansoura, Egypt. **Email:** nanees.salem@gmail.com. ORCID: 0000-0001-6783-9095.

Angi A. Alwakeel; Professor of Pediatrics, Faculty of Medicine, Mansoura University, Mansoura, Egypt**. Email:** Angiadel12363@gmail.com.

Mayada Abdel-Latif; Resident of Pediatrics, Mansoura University Children's Hospital, Faculty of Medicine, Mansoura University, Mansoura, Egypt. **Email:** doctormayada88@gmail.com.

Shaimaa R. Hendawy; Assistant Professor of Clinical Pathology, Faculty of Medicine, Mansoura University, Mansoura, Egypt. **Email**: shimaahendawy@mans.edu.eg.

Mai S. Korkor; Lecturer of Pediatrics, Faculty of Medicine, Mansoura University, Mansoura, Egypt**.** Email: mai.korkor618@yahoo.com. maikorkor@mans.edu.eg ORCID: 0000-0001-8406-6733.

<u>Corresponding Author:</u> **Mai S Korkor; MD.**

Lecturer of Pediatrics, Faculty of Medicine, Mansoura university, Mansoura, Egypt. Email: maikorkor@mans.edu.eg. ORCID: 0000-0001-8406-6733,

Another email: mai.korkor618@yahoo.com

Supplementary table: Assessment of Hardy Weinberg equilibrium (HWE) for studied genes.

		Control group (n=40)		Group with obesity (n=60)	
Frequency		Observed	Expected	Observed	Expected
Bcl1 rs41423247	CC	20	19.6	15	15.5
	CG	16	16.8	31	30.0
	GG	4	3.6	14	14.5
	P (HW)	0.763		0.794	
NR3C1 rs6198	AA	19	18.9	27	25.4
	AG	17	17.2	24	27.3
	GG	4	3.9	9	7.4
	P (HW)	0.944		0.349	

A: adenine, C: cytosine, G: Guanine, HW: Hardy Weinberg.
